# Supplementary material for: Developing the developmental origins of health and disease (DOHaD) awareness scale to assess an education module for improving dietary behavior among college students
Source: PeerJ. 2024 Dec 19;12:e18669. doi: 10.7717/peerj.18669 (PMC11663402; doi:10.7717/peerj.18669)
Supplement: Supplemental Information 13 [file peerj-12-18669-s013.docx]

|  | **A lot** | **Quite a lot** | **Somewhat** | **Not very much** | **Not at all** |
| --- | --- | --- | --- | --- | --- |
|  |  |  |  |  |  |
| Q1. How important is it to be healthy? |  |  |  |  |  |
| Q2. How important is it to eat healthy? |  |  |  |  |  |
| Q3. How important is it to exercise every day? |  |  |  |  |  |
| Q4. How much does your current diet affect your future health? |  |  |  |  |  |
| Q5. How much does your current exercise level affect your future health? |  |  |  |  |  |
| Q6. How important is nutrition during pregnancy? |  |  |  |  |  |
| Q7. How much does your social and emotional state affect your health? |  |  |  |  |  |
| Q8. How much does being breastfed affect your future health? |  |  |  |  |  |
| Q9. How much does a woman’s general health and pre-pregnancy well-being affect the fetus during pregnancy? |  |  |  |  |  |
| Q10. How much does a man’s general health and general condition before pregnancy affect the fetus during his partner’s pregnancy? |  |  |  |  |  |
| Q11. How much does a pregnant woman’s nutrition during pregnancy affect the health of the fetus? |  |  |  |  |  |
| Q12. How much does a pregnant woman’s nutrition during pregnancy affect the health of the fetus in childhood? |  |  |  |  |  |
| Q13.How much does a pregnant woman’s nutrition during pregnancy affect the health of the fetus in adulthood? |  |  |  |  |  |
| Q14. How much does a pregnant woman’s nutrition during pregnancy affect the health of the fetus in elderly? |  |  |  |  |  |
| Q15. How much does a child’s nutrition up to the age of two affect the child’s health later in life? |  |  |  |  |  |
| Q16. How much does a child’s nutrition up to the age of two affect the child’s health in adulthood? |  |  |  |  |  |
| Q17. How much does a person’s diet affect the risk of developing non-communicable diseases such as cancer, heart diseases, type 2 diabetes etc.? |  |  |  |  |  |
| Q18. How important are the first 1000 days of life in the programming of health and disease? |  |  |  |  |  |
| Q19. To what extent does inadequate and unbalanced nutrition of a pregnant woman cause permanent changes in the metabolism of fetus? |  |  |  |  |  |
| Q20. How much can a pregnant woman’s nutritional status during pregnancy affect the health of the next generations by changing gene functioning? |  |  |  |  |  |

**DEVELOPMENTAL ORIGINS OF HEALTH AND DISEASE (DOHAD) AWARENESS SCALE**
